# Time series analysis of the relationship between diarrhea in children and Rota 2 vaccine in the Fanteakwa District of the eastern region of Ghana

**DOI:** 10.1186/s12887-021-02540-3

**Published:** 2021-02-19

**Authors:** James Atampiiga Avoka, Elvis J. Dun-Dery, Issah Seidu, Armel N. E. Abou, Paul Twene, Isaac Obeng Tandoh, Frederick Dun-Dery

**Affiliations:** 1 Ghana Health Service, Birim Central Municipal Health Directorate, Box 429, Akim Oda, Ghana; 2 Department of Population and Health Research, Research Web Africa, Box 233, Sunyani, Ghana; 3 Department of Statistics, University of Ghana, P.O.Box LG 115, Legon-Accra, Ghana; 4 Institutional Public Health Unit, Eastern Regional Hospital, Koforidua, Ghana; 5 Fanteakwa North District Health Directorate, Box 60, Begoro, Eastern Region Ghana; 6 Eastern Regional Health Directorate, Ghana Health Service, Eastern Region Koforidua, Ghana; 7 Disease Control in Disadvantaged Populations, Heidelberg Institute of Global Health, Medical Faculty, Ruprecht-Karls Universität, Heidelberg, Germany

**Keywords:** Rotavirus, Diarrhea, Children, Ghana

## Abstract

**Background:**

Rotavirus is considered the main causal factor of severe gastroenteritis among infants and children globally. The association with severe rotavirus infection is mostly worse among the least developed countries, mainly due to inadequate access to medical care and poverty. This study was conducted to determine the seasonal effects in respect of diarrhea cases in children, the association between diarrhea cases and Rota2 vaccine in the Fanteakwa District of the Eastern Region of Ghana.

**Methods:**

The study compares monthly diarrhea cases against children vaccinated with Rota2 extracted from DHIMS2 spanning May 2012 to December 2017 in Fanteakwa District. A univariate association between diarrhea cases and children vaccinated with Rota 2 was conducted using the R-software version 3.4.4 with the use of forecast, tseries and TSAPred. Pearson Correlation coefficient was also computed between monthly diarrhea cases and Rota 2 as well as lagged values of Rota 2 and Diarrhea cases.

**Results:**

The study shows that February recorded the highest average number of diarrhea cases (172) over the period 2012 to 2017 with a standard deviation of 59. However, a one-way analysis of variance shows a significant difference amongst the monthly averages with an F-statistic of 0.042 and *P*-value of 0.064. It is observed that the correlations between each of the Rota2 doses and the lagged cases are positive, showing higher Rota2 doses a month ago **((*X***_***t −*** **1**_)***,*0.346** ***to*** **0.735),** two months ago **((*X***_***t −*** **2**_)***,*0.383** ***to*** **0.746**)**,** three months ago **((*X***_***t −*** **3**_**), 0.330 to 0.737)** and four months ago **((*X***_***t −*** **4**_**), 0.236 to 0.723)** are associated with lower diarrhea cases. The results also show that an increase in the previous two month’s Rota2 figures by 100 is associated with a significant decrease in the currently expected diarrhea cases by approximately 36.

**Conclusion:**

Seasonal variations exist in the occurrence of diarrhea in children, with January recording the highest number of diarrhea cases (172). There is a relationship between episodes of diarrhea in children and Rota2 (*p*-value = 0.064); thus, the more children are vaccinated with Rota2, the less diarrhea cases are recorded. Diarrhea cases in Fanteakwa district are generally low, except 2013 and 2016 where the cases are higher than the rest of the other years.

## Background

Across the globe, it is projected that about 200,000 children under 5 years of age die yearly owing to rotavirus diarrhea [14]. Discovered more than 40 years ago [8], rotaviruses are the major causative agents of acute gastroenteritis and diarrhea deaths among infants and children worldwide [13]. Group A Rotaviruses are the known major causes of diarrhea and deaths among children under five years all over the world [9] Many of such deaths occur in poor income countries [32]. Nearly a quarter of a million African children die from the deadly, dehydrating diarrhea caused by rotavirus infection every year [26] — approximately 50% of the worldwide death toll [22]. However, a major reduction of severe rotavirus (RV) diarrhea is observed in countries with high RV vaccine coverage [4]. This is due to the World Health Organization (WHO) recommendations that the rotavirus vaccine for infants should be included in all national immunization programs [21]. As a result, Ghana introduced rotavirus vaccination as part of routine immunization in 2012, and it has been shown to be effective in reducing the disease burden in children under five years [25].

Several clinical trials have been conducted especially in Africa and least developed countries, to address the issues of rotavirus cased childhood diarrhea diseases [3]. For instance, the RV1 trial study in Africa was held in two countries, South Africa and Malawi spanning from 2005 to 2007 among children aged between 5 to 10 weeks. The results from these studies indicates the potential of rota2 vaccine on childhood diarrhea in Africa. The RV5 trials were held in sub-Saharan Africa and Asia from 2007 to 2009 [33]. The one in Sub-Saharan Africa included Ghana [10], Kenya [20], and Mali [28]. This multi-Centre randomized controlled trial which was conducted showed a modest vaccine efficacy against rotavirus gastroenteritis within the first one year [28] including Ghana.

Further, studies have shown that rotavirus vaccination is effective in children, especially those under five years [25]. However, most preliminary studies have failed to assess the seasonal trend of childhood diarrhea since the implementation of the rotavirus vaccine in Ghana ([10, 14]; Ward & Bernstein, 2018). More specifically, no literature has been reported on the analysis of secondary data to show the time-series relationship between rota2 and childhood diarrhea. Therefore, the study was conducted to determine the seasonal effects of rota2 vaccine on diarrhea cases in children below five years, and the association between diarrhea cases and Rota2 vaccine in the Fanteakwa District of the Eastern Region of Ghana.

## Methods

### Data source

The data consists of monthly diarrhea cases against the number of children vaccinated with Rota 2 extracted from District Health Information Management System (DHIMS2), spanning from May 2012 to December 2017. Ghana Health Service created and scaled-up the DHIMS in 2008 with the sole aim of helping health managers and decision-makers to properly collate and analyze data at all levels of the health system. The purpose was also for planning, decision making and resource allocation within the health sector. It was implemented in all Regions and Districts throughout the country. The DHIMS2 is a free and open access software developed for health data storage and reporting. The DHIMS2 system is the National data for all health care indicators, and this is where the data were extracted. We excluded children captured in the DHIMS2 with bloody diarrhea or diarrhea caused by chronic opportunistic infections.

### Correlation and seasonal effects analysis

In order to assess the univariate association between diarrhea caused by rotavirus and number of children vaccinated with Rota 2, Pearson Correlation coefficient is computed between monthly diarrhea cases and Rota 2 as well as lagged values of Rota 2 and diarrhea cases. The lagged values are considered to know which previous month’s Rota 2 is strongly associated with diarrhea cases in the district and which lagged values of Diarrhea provides information about its future values. Diarrhea at time *t* is denoted by (***y***_***t***_) and Rota by (***x***_***t***_). Thus previous ***k*** months Diarrhea and Rota2 will thus be (***y***_***t − k***_) and (***x***_***t − k***_) respectively. Pearson correlation between any two variables (***x***_***i***_, ***y***_***i***_). is given by

$$ \boldsymbol{r}=\frac{n\sum xy-\sum x\sum y}{\sqrt{\left[n\sum {x}^2-{\left(\sum x\right)}^2\right]\left[n\sum {y}^2-{\left(\sum y\right)}^2\right]}} $$

A positive value of ***r*** shows a direct linear relationship between the paired variables and vice versa. The lagged Rota (***x***_***t − k***_) that has the highest significant correlation with the diarrhea cases will be considered as the exogenous variable in modelling. The correlation between diarrhea and its lagged value has been depicted in autocorrelation function (ACF) and partial autocorrelation (PACF) plots.

Also, a one-way ANOVA is used to check if the average diarrhea cases across the months are the same. If the number of groups to be compared is *k* and the total number of observations is *n*, the test statistic of the test is an F statistic which is

$$ F=\frac{SSB}{k-1}\div \frac{SSW}{n-k} $$

This test statistic is distributed as the F-statistic with degrees of freedom k-1 and n-k. If this test is significant at 5%, then some months have higher average cases than others, and as such, seasonal effects must be considered. Otherwise, seasonal effects are ignored. The R-software version 3.4.4 was used in the analysis of the data with the use of packages, forecast, tseries and TSAPred.

### ARIMAX Modelling

Autoregressive Integrated Moving Average (ARIMA) procedure is perhaps the most common method used in analyzing time series data. ARIMAX is just an ARIMA with an additional exogenous variable that is not the lagged values of the response variable.

According to Gujarati [35], ARIMA is preferred in situations where past values of the response variable being correlated with its future values. In order to carry out ARIMA, the data must be stationary. If not, it is differenced until it becomes stationary.

Commonly, it is stated as ARIMA (p,d,q), where *p* is the number of lags of the response variable (***y***_***t***_**)** used as regressors, *d* is the number of times (***y***_***t***_) is differenced to make it stationary, and *q* is the errors associated with forecasting the series (***y***_***t***_). The full ARIMA model when the data are stationary is given by:

$$ {\boldsymbol{y}}_{\boldsymbol{t}}={\boldsymbol{\phi}}_{\mathbf{1}}{\boldsymbol{y}}_{\boldsymbol{t}-\mathbf{1}}+{\boldsymbol{\phi}}_{\mathbf{2}}{\boldsymbol{y}}_{\boldsymbol{t}-\mathbf{2}}+\dots +{\boldsymbol{\phi}}_{\mathbf{1}}{\boldsymbol{y}}_{\boldsymbol{t}-\boldsymbol{p}}+{\boldsymbol{\varepsilon}}_{\boldsymbol{t}}+{\boldsymbol{\theta}}_{\mathbf{1}}{\boldsymbol{\varepsilon}}_{\boldsymbol{t}-\mathbf{1}}+{\boldsymbol{\theta}}_{\mathbf{2}}{\boldsymbol{\varepsilon}}_{\boldsymbol{t}-\mathbf{2}}+\dots +{\boldsymbol{\theta}}_{\mathbf{1}}{\boldsymbol{\varepsilon}}_{\boldsymbol{t}-\boldsymbol{q}} $$

The Box-Jenkins procedure is used to estimate values of p,d,q. The Box-Jenkins method involves the model specification, parameter estimation and diagnostics checking. The response variable ***y***_***t***_ is assumed to be normally distributed as such, the maximum likelihood estimation method is used to find the parameter ***ϕ*** and ***θ***. However, the auto Arima function in the forecast R-package uses the Box-Jenkins based algorithm to get the best estimates for p,d and q as well their associated parameter estimates [36].

By adding an exogenous variable to the model (lagged Rota, ***x***_***t − k***_), the model becomes ARIMAX, given by

$$ {\boldsymbol{y}}_{\boldsymbol{t}}={\boldsymbol{\phi}}_{\mathbf{1}}{\boldsymbol{y}}_{\boldsymbol{t}-\mathbf{1}}+{\boldsymbol{\phi}}_{\mathbf{2}}{\boldsymbol{y}}_{\boldsymbol{t}-\mathbf{2}}+\dots +{\boldsymbol{\phi}}_{\mathbf{1}}{\boldsymbol{y}}_{\boldsymbol{t}-\boldsymbol{p}}+{\boldsymbol{\varepsilon}}_{\boldsymbol{t}}+{\boldsymbol{\theta}}_{\mathbf{1}}{\boldsymbol{\varepsilon}}_{\boldsymbol{t}-\mathbf{1}}+{\boldsymbol{\theta}}_{\mathbf{2}}{\boldsymbol{\varepsilon}}_{\boldsymbol{t}-\mathbf{2}}+\dots +{\boldsymbol{\theta}}_{\mathbf{1}}{\boldsymbol{\varepsilon}}_{\boldsymbol{t}-\boldsymbol{q}}+\boldsymbol{\beta} {\boldsymbol{x}}_{\boldsymbol{t}-\boldsymbol{k}} $$

To test the effect of each of the regresses on the monthly diarrhea cases, a hypothesis of the corresponding parameter is tested if it is significantly different from zero. The test statistic in this case is

$$ \boldsymbol{Z}=\frac{{\boldsymbol{\alpha}}_{\boldsymbol{i}}^{\ast}}{\boldsymbol{s.e}\left({\boldsymbol{\alpha}}_{\boldsymbol{i}}^{\ast}\right)} $$

Where ${\boldsymbol{\alpha}}_{\boldsymbol{i}}^{\ast}$ is the regression parameter estimate of the regression ***w***_***i***_ and $\boldsymbol{s}.\boldsymbol{e}\left({\boldsymbol{\alpha}}_{\boldsymbol{i}}^{\ast}\right)$ is the corresponding standard error. The statistic follows the standard normal distribution.

### Validation of model

In order to validate the model, the data set was divided into two sets, the training set from May 2012 to December 2016, whereas January 2017 to December 2017 is used to validate the model. This is to ensure the consideration and inclusion of proportional diarrhea cases as well as to validate the accuracy of data reporting in DHIMS over the study period. If the actual data values fall within the 95% confidence interval of the predicted estimates, the model was deemed robust. Also, the Root Mean Square Error (RMSE) and the Mean Absolute Percentage Error (MAPE) was reported.

## Results

### The trend of diarrhea cases

**Fig. 1:**
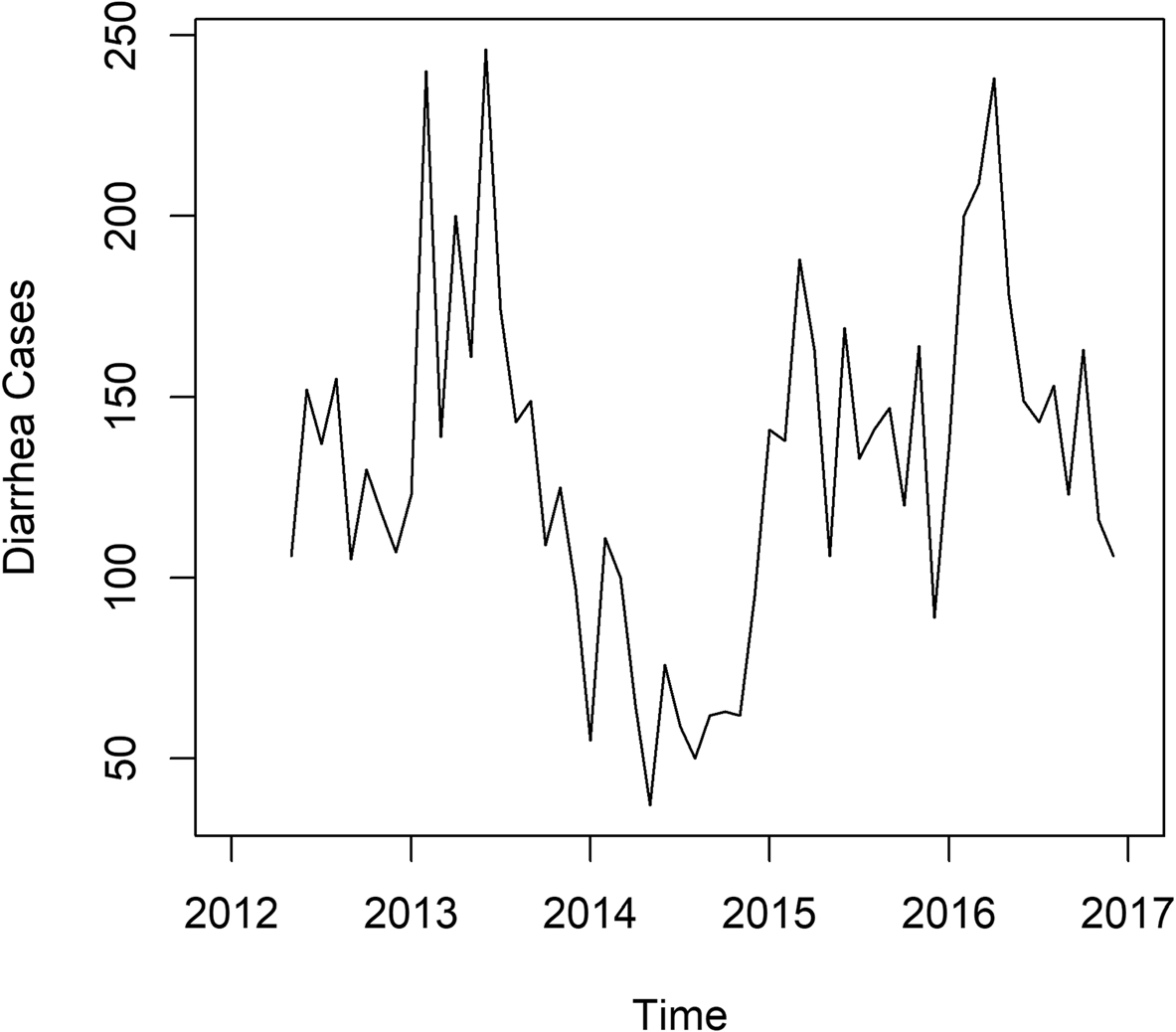
Time Series Plot of Diarrhea Cases from 2012 to 2017

From Fig. 1, it is noticed that the diarrhea cases are generally low except 2013 and 2016 where the cases are higher than the rest of the other years.

### Correlation between diarrhea cases and its lagged values

**Fig. 2:**
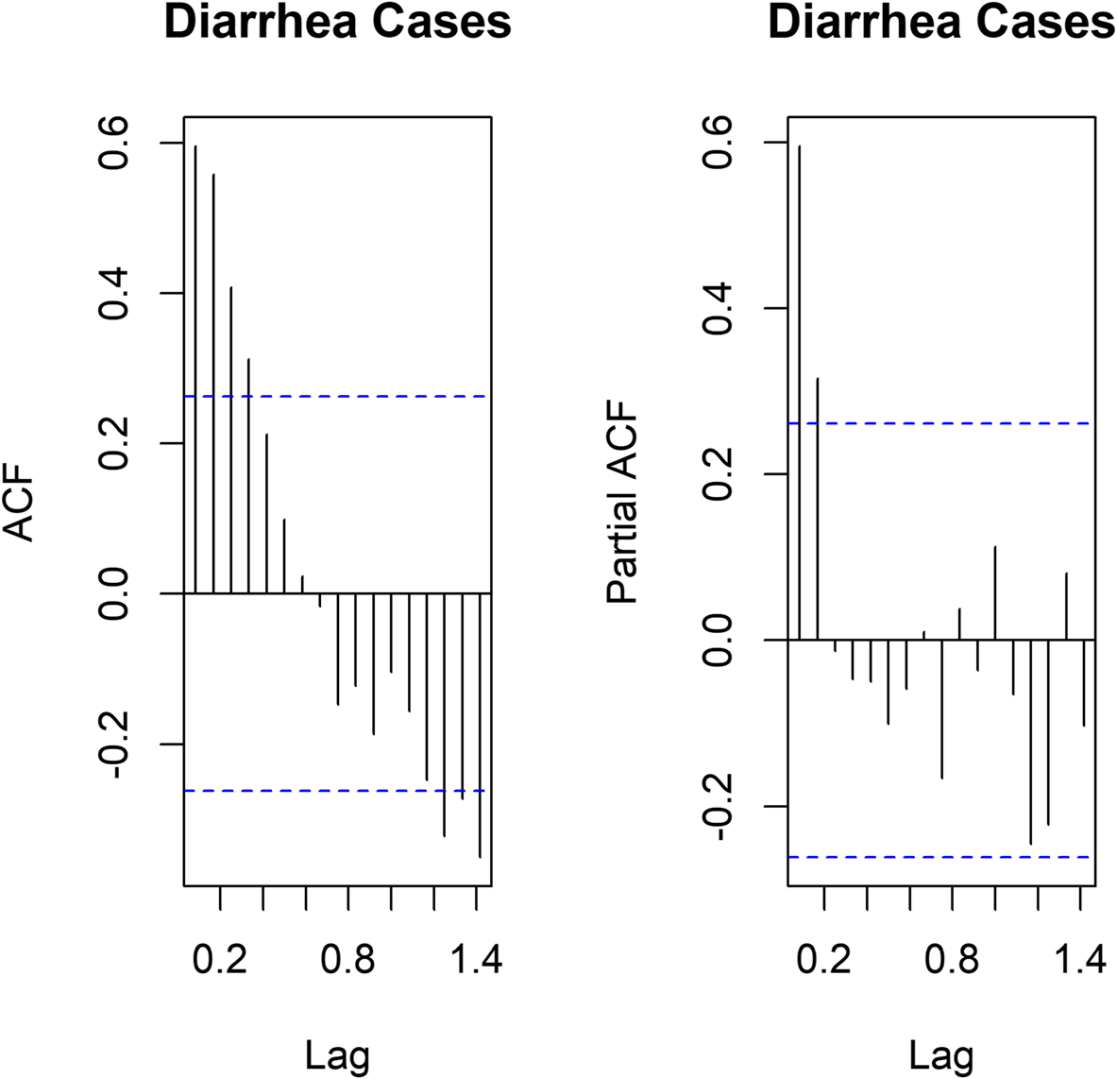
ACF and PACF plots of Diarrhea Cases

From the ACF and PACF plots, up to five lags are negatively associated with diarrhea cases, and only two lags are significantly associated, controlling for all the other lag values. This means that the first two lag values (previous two months) contain the information needed to predict diarrhea cases (Fig. 2).

From Table 1, February recorded the highest average number of diarrhea cases over the period 2012 to 2017 to be 172 cases with a standard deviation of 59. On the other hand, December recorded the lowest number of average cases of 99, with a standard deviation of 8 cases. However, a one-way analysis of variance to compare the monthly averages gave an F-statistic of 0.042 with a corresponding *p*-value of 0.064 showing significant differences amongst the monthly averages. This implies that there are seasonal effects in diarrhea over the different months under study.

**Table 1:** Seasonal Effect of Diarrhea Cases

Month	***n***	***mean***	***Std. Dev***
January	4	114	40
February	4	172	59
March	4	159	49
April	4	167	74
May	5	118	55
June	5	158	61
July	5	129	42
August	5	128	44
September	5	117	36
October	5	117	36
November	5	117	36
December	5	99	8
F = 0.042	*p*-value = 0.064		

Source: Field data

### Selecting a covariate from ROTA

From Table 2 above, it is noticed that the correlations between each of the Rota2 doses and the lagged cases are protective of the outcome of rotavirus causing diarrhea among under five, showing lower Rota2 cases a month ago (***X***_***t −*** **1**_), two months ago (***X***_***t −*** **2**_)**,** three months ago (***X***_***t −*** **3**_) and four months ago (***X***_***t −*** **4**_), are associated with lower diarrhea cases. The correlation peaks at lag 2 where it is 0.383 showing that the previous two months Rota is to be chosen as the covariate in predicting Diarrhea cases in the district.

**Table 2:** Correlations between Diarrhea and Lagged Rota2 Cases

	***Y***_***t***_	***X***_***t***_	***X***_***t −*** **1**_	***X***_***t −*** **2**_	***X***_***t −*** **3**_
***X***_***t***_	0.225371				
***X***_***t −*** **1**_	0.346840	0.735149			
***X***_***t −*** **2**_	0.383489	0.625716	0.745684		
***X***_***t −*** **3**_	0.330436	0.652169	0.620834	0.737329	
***X***_***t −*** **4**_	0.236041	0.625549	0.632542	0.593118	0.723181

Source: Field data

### Arimax model for diarrhea

Using the auto.arima command in the forecast package in R with lagged Rota as an exogenous variable, the ARIMA (2,0,0) is chosen as the best fit model for the data (see Table 3).

**Table 3:** Arimax Coefficients

Variable	Coefficient	Std. Error	***p***-value	RMSE	MAPE
***Y***_***t −*** **1**_	0.389	0.131	0.003	28.05849	19.26922
***Y***_***t −*** **2**_	0.352	0.131	0.007		
***X***_***t −*** **2**_	0.359	0.057	0.000		

From Table 3, the fitted model that estimates current diarrhea cases (***Y***_***t***_) is given by

$$ {\hat{\boldsymbol{Y}}}_{\boldsymbol{t}}=\mathbf{0.389}{\boldsymbol{Y}}_{\boldsymbol{t}-\mathbf{1}}+\mathbf{0.352}{\boldsymbol{Y}}_{\boldsymbol{t}-\mathbf{2}}+\mathbf{0.359}{\boldsymbol{X}}_{\boldsymbol{t}-\mathbf{2}} $$

The model shows that controlling for the lag values of diarrhea, a decrease in the previous two month’s Rota2 figures is associated with a significant decrease in the currently expected diarrhea cases by approximately 36. The model predicts diarrhea cases with an average standard error of 28 cases with a Mean average error of prediction to be 19%.

### Validation of model and predicting diarrhea cases

**Fig. 3:**
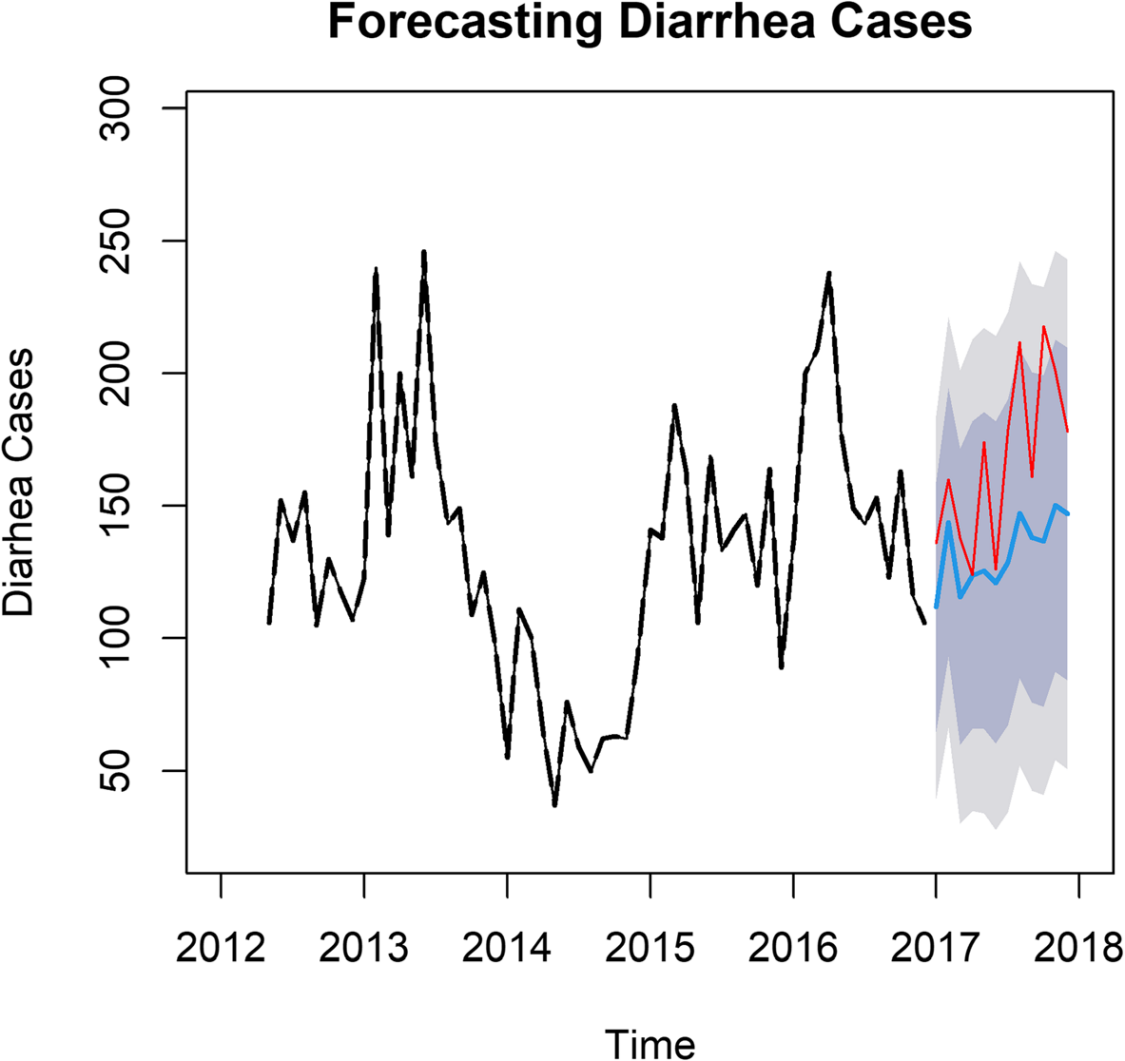
Comparing predicted and actual values of diarrhea cases in 2018

Using the TSAPred package, a plot of the predicted diarrhea cases in blue is given in Fig. 3 together with their 90 and 95% confidence intervals in deep grey and light grey, respectively. The actual values of diarrhea cases plotted in red are juxtaposed on the plot to validate the model. It is realized that though the actual values are generally higher than the predicted values of diarrhea, they fall within the 95% confidence intervals of the corresponding estimates. This implies that the bounds of the 95% confidence interval, especially the upper bound do not wrongfully predict the actual diarrhea cases. Figure 3 further suggest that diarrhea case varies within years depending on the efficacy and administration of rota2 vaccine. In general, cases are lower in most years between 2012 and 2017. It is predicted to rise steadily in 2018 but likely to fall in subsequent years, subject rota2 vaccine administration. Predicted *p*-values for 2018 indicates that lower diarrhea cases will be recorded as *p*-values shows a positive association between rota2 vaccine and childhood diarrhea.

## Discussion

The results of this study reveal that there are seasonal changes in the diarrhea cases generated which contradicts the study conducted by [23]. Similar studies, conducted in China however, recorded a strong seasonal pattern with peaks in November–December and in July–August [15], which agrees with the current study. Possible reasons for the variations reported in the China study and that of our study are the difference in vaccination periods and strict implementation of immunization programs, especially in Ghana, where populations are more vulnerable compared to China’s situation.

Meanwhile, according to Armah et al. [37], the vaccine efficacy against severe rotavirus gastroenteritis was reported to be 39.3% in certain parts of Africa but up to 98% in the America and Europe [38]. This shows that there is a huge percentage gap in the rotavirus disease situation in parts of Africa, compared to the America and Europe. This could be influencing the varied efficacy of the vaccine from one area to the other. The situation in Africa or Ghana could be due to certain factors such as high prevalence of under nutrition, high prevalence of underweight, and high prevalence of unusual rotavirus strains as reported by CDC and antigen diversity [39, 40]. Early development of microbiome in infant guts overlaps with that of the immune response. The initial stage of microbial and immunological development timeously matches that of the first immunization [19]. The first effect may affect vaccine oriented immunity. Studies show that too much pathogenic load affects the potency of oral vaccines especially in low and middle income countries [12] which also gives more evidence from other cohort studies [18].

Like a study conducted in the United States of America [34] and other places where there was a decrease in the number of hospital admissions for all-cause diarrhea and rotavirus, our study recorded a significant decrease in the number of reported cases of all-cause diarrhea in all health facilities within the district for six (6) consecutive years, 2012–2017. Conversely, the trend of increasing diarrhea cases among under-five has been reported in several studies after the implementation of the rota vaccine [3, 24, 30]. In countries with increase reported cases, healthcare systems are becoming stronger, and resources are being directed towards the prevention of conditions such as diarhhea. This may explain the difference in reported cases in both studies. However, the decreasing trend of rotavirus diarhea is reported and sustained in several developed countries due to advanced health care services on maternal and child care [31].

The study reveals a correlation between Rota2 coverage, including the lagged cases, positively showing an association with lower diarrhea cases. The implication is that, the higher the Rota2 doses given for a month, the lower the diarrhea cases recorded for that month. In line with this finding similar studies conducted in Brazil on diarrhea incidence among rotavirus vaccinated infants, reported a progressive reduction in the proportion of diarrhea cases since the introduction of rota vaccine [27]. Conversely, studies conducted in Yemen on all-cause rotavirus cases, reported a fluctuating incidence of hospitalized diarrhea cases due to rotavirus [3] and is further reported high among Croatian population of under-five, following the immediate implementation of rota2 vaccine [32]. This finding suggests that the attenuating effect of the vaccine may be responsible for the variations in diarrhea cases. Additionally, cost, vaccine availability, cold chain management and vaccine monitoring challenges may also affect the efficacy of vaccination in different countries, especially developing countries [5]. Several findings reveal that live oral vaccines including that of rotavirus have had low performance in low and middle-income countries including Ghana ([6, 7, 16, 34, 41]. In order to completely eradicate vaccine preventable diseases like rota virus diarhea among children in developing countries, strict vaccine management and monitoring are required throughout the cold-Chain management.

The model in this study shows that controlling for the lag values of diarrhea, a decrease in the previous two month’s Rota2 coverage is associated with a significant decrease in the currently expected diarrhea cases by approximately 36. This is in line with studies conducted among young children to assess the seasonal association between diarrhea, Tropheryma whipplei and rota vaccine, which reveals decreasing seasonal variations in diarrhea cases between different months and years [17]. Again, our study anticipates a decrease in diarrhea cases, just like studies in northwestern Ethiopia, which reported decreasing trends of diarrhea cases as a result of breastfeeding and rota vaccine implementation program [29]. Large multi-site randomized controlled trials in low-income settings such as Ghana, have demonstrated vaccine efficacy against severe rotavirus gastroenteritis in the first year of life [28] and difference in the incidence of diarrhea cases are sometimes attributed to low health budgets [1].

The current study predicts diarrhea cases with an average standard error of 28 cases with a Mean average percentage error of 19%. In other studies, contradictory findings were reported with an average of 42.9% [3]. It is worth noting that poor quality of care and ill-preparedness on the part of providers to deliver timely, sensitive care to children with diarrhea can lead to increasing trends in diarrhea cases [2]. In respect of the above findings, it could mean that the type of rotavirus vaccine used in our part of the world and Fanteakwa District, in particular, may be the exact vaccine meant to reduce diarrhea-related hospital admissions. If rotavirus is the commonest root cause of severe diarrhea in children globally, as reported [11], then the substantial decrease in the number of reported cases of all-cause diarrhea [23] in children under five in the health facilities within our study area is expected.

## Conclusion

Seasonal variations do influence the occurrence of diarrhea in children under five years. There is a relationship between episodes of diarrhea in children and Rota2; thus, the more children are vaccinated with Rota2, the less diarrhea episodes will occur among them. Whiles it is imperative for health care providers to enhance maternal education on vaccination, ensuring continuous rota2 vaccination for all eligible children is critical to ending rotavirus related diarrhea.

### Limitations

Data were extracted directly from the DHIMS2 platform, and therefore, any data inaccuracy or error arising out of the system cannot be ascertained. Secondly, the efficacy and potency of the vaccines that were given to the children in this study could not be authenticated by the study. Additionally, completeness of reporting, timeliness of reporting, and data inconsistencies or data quality issues are possible data issues. Data captured in the DHIMS2 system are not disaggregated into the various age groups, therefore analysis involving the different age groups was impossible. However, in anticipation of these data entry errors, the data were captured ranging from 2012 to 2017 and divided into training set from May 2012 to December 2016, whereas January 2017 to December 2017 is used to validate the accuracy of data and nullify the effects of outliers. Therefore, data quality issues cannot be attributable to the results shown in this study.

## Data Availability

For confidentiality reasons, we are unable to share these data publicly, but the corresponding author can be contacted for further information.

## Abbreviations

DHIMS2: District Health Information Management System 2
GHS: Ghana Health Service
ACF: Auto-correlation Function
PACF: Partial Autocorrelation
ANOVA: Analysis of variance
ARIMA: Autoregressive Integrated Moving Average
ARIMAX: ARIMA with additional exogenous variable that is not the lagged values of the response variable
RMSE: Root Mean Square Error
MAPE: Mean Absolute Percentage Error
CDC: Centers for Disease Control; WHO: World Health Organization
